# Regulation and analysis of Simiao Yong’an Decoction fermentation by *Bacillus subtilis* on the diversity of intestinal microbiota in Sprague-Dawley rats

**DOI:** 10.14202/vetworld.2024.712-719

**Published:** 2024-03-25

**Authors:** Zhen Yang, Keyuan Chen, Yu Liu, Xuehong Wang, Shengyi Wang, Baocheng Hao

**Affiliations:** Key Laboratory of New Animal Drug Project, Gansu Province, China; Key Laboratory of Veterinary Pharmaceutical Development, Ministry of Agriculture and Rural Affairs, Lanzhou Institute of Husbandry and Pharmaceutical Sciences of Chinese Academy of Agriculture Sciences, Lanzhou, China

**Keywords:** *Bacillus subtilis*, fermentation, intestinal microbiota, probiotics, Simiao Yong’an Decoction

## Abstract

**Background and Aim:**

Simiao Yong’an decoction (SYD) is a classic traditional Chinese medicine (TCM) prescription that has the effects of clearing heat, detoxifying, promoting blood circulation, and relieving pain. In this study, we investigated the effect of SYD on the diversity of intestinal microbiota after fermentation by *Bacillus subtilis*.

**Materials and Methods:**

SYD was fermented using *B. subtilis*. Female Sprague-Dawley rats were randomly divided into the following four groups with six rats in each group: Negative sample group (NS), water exaction non-fermentation group (WE), *B. subtili*s group (BS), and fermentation liquid group (FL). All rats were orally administered for 14 days. High-throughput Illumina sequencing was used to analyze 16S rRNA expression in rat fecal samples.

**Results:**

A total of 2782 operational taxonomical units (OTUs) were identified in this study, and 634 OTUs were shared among all samples. Bacteroidetes (28.17%–53.20%) and Firmicutes (48.35%–67.83%) were the most abundant phyla identified among the four groups. The abundance of Escherichia and Alistipes was lower in the FL group than in the NS group, whereas the abundance of Bifidobacteria and Lactobacillus was increased in the FL group (p < 0.05). The abundance of Bifidobacterium was significantly upregulated in the FL group compared with the WE and BS groups (p < 0.05).

**Conclusion:**

After fermentation, SYD had a significantly better effect than SYD or *B. subtilis*. SYD significantly promoted the growth of intestinal probiotics, inhibited the growth of pathogenic bacteria, and maintained the balance of intestinal microbiota in SD rats. This study provides new insights into the development and use of SYD.

## Introduction

The intestinal microbiota comprises diverse symbiotic bacterial species that play different physiological and molecular functions in the intestinal tract of animals [1]. Although intestinal microbiota has been proven to help resist the colonization and invasion of pathogens [2], structural changes are also closely related to many metabolic diseases [3]. Dysbiosis, that is, perturbations in the composition of the intestinal microbiota, has been reported to be associated with a higher risk of developing specific diseases [4], including chronic gastrointestinal inflammatory diseases [5], irritable bowel syndrome [6], diarrhea [7], diabetes [3], and obesity [8]. The maintenance of intestinal microbiota structure and balance was significantly affected by the daily diet of animals. Therefore, a critical way to ensure animal health is to avoid diseases that affect the stability of the intestinal microbiota [9, 10].

In China, “medicine food homology” (MFH) is a concept from which food and traditional Chinese medicine (TCM) originate simultaneously. It scientifically combines the functions of food and medicine, thereby exerting disease prevention, treatment, and other health-care functions of MFH materials in addition to their nutritional value [11]. Numerous herbal or functional products have been used in Asia for millennia and have their origins in TCM. The popularity of TCM has increased worldwide in recent years without signs of weakening in the short term. Beneficial products, such as herbal foods, dietary supplements, and functional foods, described by TCM help to correct metabolic disturbances or imbalances of organisms [12]. Simiao Yong’an decoction (SYD) comprises Flos Lonicerae japonicae (*Lonicera japonica* Thunb., Jinyinhua), Radix Scrophulariae (*Scrophularia ningpoensis* Hemsl., Xuanshen), Angelicae Sinensis (*Angelica sinensis* [Oliv.] Diels, Danggui), and Glycyrrhizae Uralensis (*Glycyrrhiza uralensis* Fisch., Gancao) in a 3:3:2:1 proportion by weight [13]. According to the classic TCM Yan Fang Xin Bian, SYD has the effects of clearing heat, detoxifying, promoting blood circulation, and relieving pain. It has also been used to treat dislocations [14]. At present, SYD shows good efficacy for the treatment of cardiovascular-related diseases, and it has been widely studied for the treatment of hypertension, myocarditis, and other diseases [15–17].

Certain undesirable compounds in food and medicine are commonly broken down and induced efficient microbial transformation through fermentation [18, 19] to improve their potential. The previous research showed that natural ingredients, including phytosterol isoflavones and saponins, can be modified by fermentation processes that enhance their biological activities [20]. It is a natural and safe way to produce beneficial effects by fermented probiotic strains. Fermentation using probiotics is a natural and safe way to add a barrier against microbial infection [21, 22]. Immunity, intestinal integrity, and intestinal microbiome disorders have been reported to be improved by probiotic fermentation broth of TCM [23].

*Bacillus subtilis* is a probiotic that has recently been widely used in food, medicine, feed additives, and other fields [24–26]. When entering the animal intestine, it can consume a large amount of free oxygen, reduce the intestinal oxygen concentration and redox potential, thereby improving the growth environment of anaerobic bacteria such as *Lactobacilli* and *Bifidobacteria*, maintaining the stability of the internal environment of the intestinal microbiota, enhancing the resistance of the animal body, and reducing the occurrence of animal gastrointestinal diseases [27]. Wang *et al*. [28] fermented *Astragalus membranaceus* with *B. subtilis* and revealed its therapeutic effect on hyperuricemia by regulating the intestinal microbiota through multi-omics analysis. In addition, *B. subtilis* promotes the development of animal immune organs and early maturation of the immune system, thereby enhancing the immunity of animals [29, 30].

On the basis of these findings, it can be concluded that the use of probiotics to ferment TCM can enhance the biological activity of TCM and maintain the stability of the intestinal microbiota [31]. At present, there are few reports on SYD fermented by probiotics. SD rats were orally administered four different diets (negative control, SYD non-fermentation extract, *B. subtilis*, and SYD fermentation broth) in this study. We collected and analyzed the 16S rRNA genes of fecal samples to compare the differences in the fecal microbial communities to better understand the influences of the four diets on the intestinal microbiota.

This study aimed to explore the influence of SYD fermentation broth on the diversity of intestinal microbiota and lay a theoretical foundation for its clinical application and development.

## Materials and Methods

### Ethical approval

Female Sprague-Dawley (SD) rats were obtained from the Lanzhou Veterinary Research Institute of the Chinese Academy of Agricultural Sciences. All rats in this study were cared for according to the specifications of the Ethics Committee of the Lanzhou Institute of Husbandry and Pharmaceutical Sciences of the Chinese Academy of Agriculture Sciences for the Care and Use of Laboratory Animals (Approval No. 2018-094).

### Study period and location

This study was conducted from October 2018 to December 2020 at the Lanzhou Institute of Husbandry and Pharmaceutical Sciences of the Chinese Academy of Agriculture Sciences, China.

### Medicinal materials

We purchased Flos Lonicerae Japonicae, Radix Scrophulariae, Angelicae Sinensis, and Glycyrrhizae Uralensis from Min County, Gansu Province, China. *B. subtilis* was purchased from the China Centre of Industrial Culture Collection (CICC 10089). Glucose, peptone, and yeast extract powder were obtained from Aobox Biotechnology (Beijing, China). The E.Z.N.A.^®^Stool DNA Kit was purchased from Omega Inc., USA. Other chemical reagents, including K_2_HPO_4_, MgSO_4_, and CaCO_3_, were purchased from local suppliers. All chemical reagents used were of analytical grade.

### Preparation of the SYD

Flos Lonicerae Japonicae, Radix Scrophulariae, Angelicae Sinensis, and Glycyrrhizae Uralensis were accurately weighed in a ratio of 3:3:2:1, evenly crushed, vortex mixed, and sieve filtered to prepare SYD.

### Preparation of SYD and *B. subtilis* fermentation broth

The fermented liquid medium (FLM) was prepared in 250-mL flasks containing 4% dried SYD powder, 1% glucose, 2% peptone, 0.3% yeast extract powder, 0.07% MgSO_4_, 0.05% K_2_HPO_4_, 0.02% CaCO_3_, and 100 mL of distilled water (initial pH 7.2). The stock culture was incubated in Luria broth (LB) at 160 rpm and 37°C in a shaker for 24 h until the logarithmic phase. 3% (v/v) pre-cultured *B. subtilis* (3.6 × 10^8^ colony-forming unit [CFU]/mL) was placed in a shaker at 160 rpm and 37°C for 48 h to perform liquid fermentation and bacterial culture (LB) to obtain the fermentation product (*B. subtilis*, 5.4 × 10^8^ CFU/mL). Fermented products were freeze-dried and stored for further use. SYD (unfermented product) was obtained in the same manner, but no bacteria were used in this process.

### Experimental animals

Female SD rats weighing 180–220 g were randomly divided into the following four groups (n = 6) after 1-week adaptive feeding. The negative sample group (NS) was administered 3 mL of normal saline (0.9%), the SYD fermentation liquid group (FL) was administered SYD fermentation broth by gavage with 3 mL of normal saline at a dose of 2.1 g/kg, the SYD water exaction non-fermentation group (WE) was administered SYD unfermented product by gavage with 3 mL of normal saline at a dose of 2.1 g/kg, and the *B. subtilis* group (BS) was administered *B. subtilis* (5.4 × 10^8^ CFU/rat) by gavage with 3 mL of normal saline. All rats were orally administered daily, and the experiment lasted for 2 weeks. On average, 100–200 mg of fecal samples were collected from each rat and processed using sterile forceps. For DNA extraction, the collected samples were immediately frozen to –80°C.

### DNA extraction

The E.Z.N.A.®Stool DNA Kit (D4015, Omega Inc.) was used to extract DNA from the fecal samples according to the manufacturer’s instructions. The extracted total DNA was eluted with 50 μL elution buffer and stored at –80°C for polymerase chain reaction analysis using LC-Bio (Hangzhou, China). About 1.2% of agarose gel electrophoresis was used to confirm the isolation of DNA.

### Analyses of intestinal microbiota

Before sequencing, a set of primers targeting the 16S rRNA gene region was used to amplify the 16S rDNA V3–V4 region of each sample. The NEB Next Ultra DNA Library Prep Kit for Illumina (NEB, USA) was used to generate the sequencing libraries and add the index codes, as recommended by the manufacturer. The Qubit@ 2.0 Fluorometer (Life Technologies, CA, USA) and Agilent Bioanalyser 2100 system were used to assess library quality. Finally, library sequencing was performed on the Illumina MiSeq platform (Illumina, San Diego, CA, USA) with 300 bp paired-end reads [32].

### Analysis of bioinformatics

The Illumina MiSeq platform was used to sequence the samples according to the manufacturer’s instructions. Paired-end reads were assigned and truncated based on the unique barcode of the samples by cutting off the barcode and primer sequences. Fast Length Adjustment of SHort reads was used to merge the paired-end reads. Clean high-quality tags were obtained by quality filtration of raw tags using fqtrim (v 0.94) (https://ccb.jhu.edu/software/fqtrim/) under specific filtering conditions. We used Vsearch software (v 2.3.4) (https://github.com/torognes/vsearch) to filter the chimeric sequence. Vsearch (v 2.3.4) was used to assign sequences with ≥97% similarity to the same operational taxonomic units (OTU). Each OUT included selected representative sequences that were then assigned with taxonomic data through the Ribosome Database Project classifier, available online: http://rdp.cme.msu.edu/index.jsp. Mafft software (v 7.310) (https://mafft.cbrc.jp/alignment/software/) was used to determine the differences of dominant OTUs among different groups, thereby studying the phylogenetic relationships among different OTUs based on multiple sequence alignment. A standard sequence number corresponding to the fewest sequences of the sample was used to normalize OTU abundance information. The complexity of sample OTU diversity was analyzed by alpha diversity through indices in the samples that included Chao1 and the observed OTUs, both of which were calculated using QIIME (v 1.8.0) (http://qiime.org/home_static/dataFiles.html). Differences in sample OTU complexity were evaluated by beta diversity analysis. Principal component analysis (PCA) was used to calculate beta diversity, whereas QIIME (v 1.8.0) was used to calculate cluster analysis [33].

### Statistical analysis

Experimental data were analyzed by one-way analysis of variance (ANOVA) in Statistical Package for the Social Sciences (SPSS) 23.0 for Windows (SPSS Inc., Chicago, IL, USA). Least significant difference multiple comparisons were used to test the significance of the differences among the four groups. p < 0.05 was considered statistically significant, and the data analysis results are expressed as the mean ± standard deviation (SD).

## Results

### Relative abundance and diversity of the OTUs

**Figure-1:**
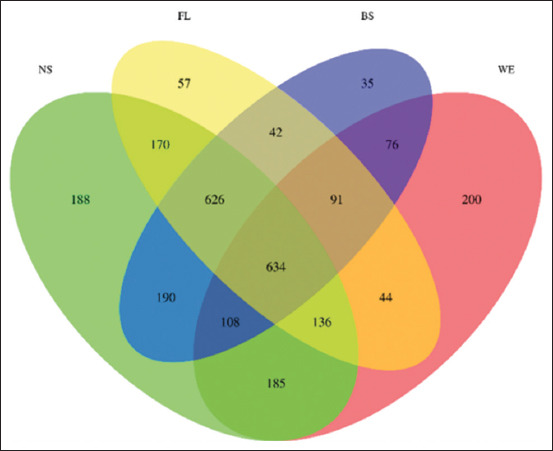
Relative abundance and diversity of operational taxonomical units in fecal samples from each group.

The OTUs shared by the samples are clearly illustrated in the Venn diagram (Figure-1). According to the analysis results, 2782 OTUs were detected in all samples, but only 634 OTUs were shared in the total abundance. In our study, 2237, 1800, 1802, and 1474 OTUs were obtained from samples from the four groups (NS, FL, BS, and WE).

There were 2237, 1800, 1802, and 1474 OTUs in the NS, FL, BS, and WE samples, respectively. The overlapping OTUs included 1566 OTUs shared between the NS and FL samples, 1558 OTUs shared between the NS and BS samples, 1063 OTUs shared between the NS and WE samples, 1393 OTUs shared between the FL and BS samples, 905 OTUs shared between the FL and WE samples, 1802 OTUs shared between the BS and WE samples, 1260 OTUs shared among the NS, FL, and BS samples, 770 OTUs shared among the NS, FL, and WE samples, 742 OTUs shared among the NS, BS, and WE samples, 725 OTUs shared among the FG, PG, and WG samples. The total richness of all samples was 2782.

### Variation in alpha diversity

**Figure-2:**
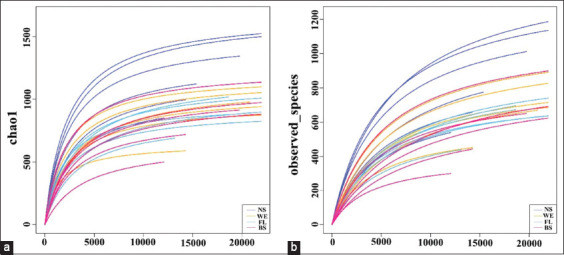
Comparison of the diversity indices of different methods. (a) Rarefaction curves based on Chao1. (b) Observed operational taxonomical units index.

The indexes of Chao1 (Figure-2a) and observed OTUs (Figure-2b) were used to assess alpha diversity, and all indicators showed the same trend. As shown in Figure-2a, the rarefaction curves of Chao1 indexes almost reached the saturation platform in four groups at 10,000 sequencing depth. The abundance of microbial communities was lowest in the BS samples (p < 0.05) and highest in the NS samples. These results indicated that the NS samples had the highest microbial community diversity (p < 0.05) (Table-1).

**Table-1:** The impact of different treatments on biodiversity.

Groups	Observed OTUs	Chao1
NS	800.00 ± 183.23	1187.45 ± 241.91
WE	611.50 ± 107.64^a^	888.20 ± 180.95^a^
FL	560.17 ± 64.65^b^	814.63 ± 95.89^b^
BS	523.00 ± 156.67^c^	813.77 ± 167.14^c^

The results are expressed as the mean ± standard deviation (n = 6). Different letters indicate significant differences (p < 0.05) that compared with NS group. OTUs=Operational taxonomical units, NS=Negative sample group, WE=Water exaction non-fermentation group, BS=*Bacillus subtilis* group, FL=Fermentation liquid group

### Variation in beta diversity

**Figure-3:**
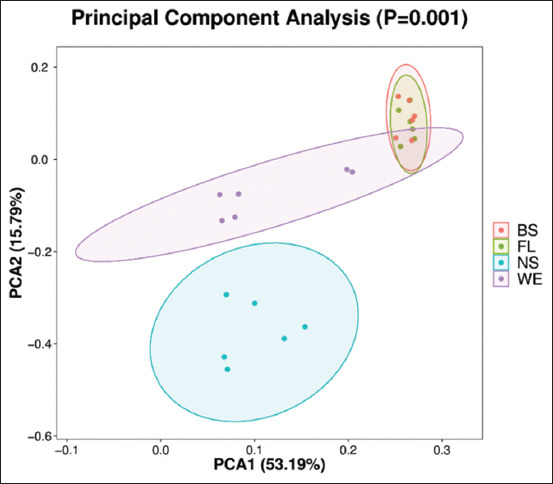
Principal component analysis plots according to the four groups’ unweighted UniFrac distance matrices.

The differences in the fecal microbial populations among the four groups could be conveniently observed through PCA plots that were performed according to the unweighted UniFrac distance matrix. Significant differences among the fecal samples of the NS, WE, and BS or FL groups indicated that different diet patterns had a significant impact on the microbial community in the fecal samples of rats (Figure-3).

### Structure of microbial communities at the phylum, family, and genus level

**Figure-4:**
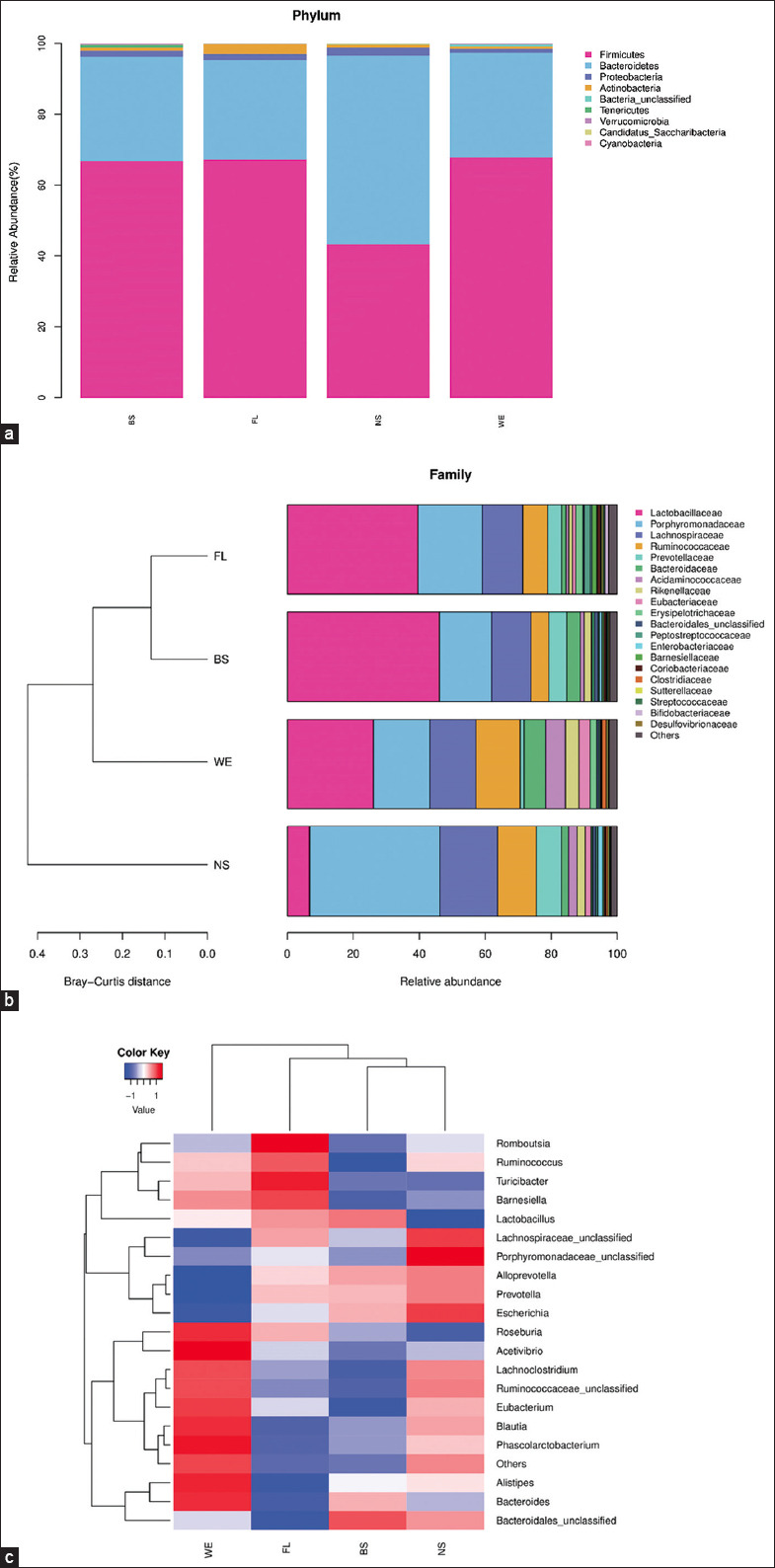
(a) Top phyla’s relative abundance. (b) Bar chart of the top families in samples. (c) Heatmap of each genus in samples.

As illustrated in Figure-4, more than nine different phyla were identified based on the classification of sequences from the samples in the study of taxonomic composition. *Bacteroidetes* (28.17%–53.20%) and *Firmicutes* (48.35%–67.83%) were the most abundant phyla identified in the four groups (Figure-4a). *Actinobacteria*, *Tenericutes*, *Proteobacteria*, *Verrucomicrobia*, *Cyanobacteria*, and two unclassified bacterial phyla showed relatively low abundances.

To better understand the structure of the rat fecal microbiota, fecal sample analysis was performed at the family level. As shown in (Figure-4b), Lactobacillaceae accounted for 29.65%, followed by Porphyromonadaceae and Lachnospiraceae. Lactobacillaceae accounted for 46.13%, followed by Porphyromonadaceae and Lachnospiraceae, which were the predominant family in samples of the BS group. The abundance of Lachnospiraceae in samples from the BS group was significantly higher than that in the other groups (p < 0.05), whereas the abundance of Lachnospiraceae in samples from the NS group was significantly lower than that in the other groups (p < 0.05). Moreover, an unclassified sequence named Bacteroidales was found at the family level, which comprised 0.99, 0.62, 0.33, and 1.24% of the samples of the NS, WE, FL, and BS groups, respectively.

The different genera in the samples are illustrated through the heatmap (Figure-4c). Lactobacillus content was highest in the BS group (46.13%), followed by the FL (39.63%), WE (26.11%), and NS (6.75%) groups (p < 0.05). Alistipes relative abundance was slightly decreased (BS, 2.11%; FL, 1.11%; NS, 2.39%) (p > 0.05) after treatment. In addition, the abundance of Escherichia in the samples of the NS group (1.46%) was above that of the WE (0.16%), BS (0.80%), and FL (0.44%) groups (p < 0.05), whereas the abundance of Bifidobacterium in the samples of the NS group (0.06%) was below that of the WE (0.11%), BS (0.16%), and FL (1.07%) groups (p < 0.05).

## Discussion

Intestinal microbiota is essential for the absorption and transformation of nutrients in the animal’s daily diet, which organically maintains the entire homeostatic network of the body through its pivotal role in the control of bacteria and inflammation [34]. Natural products have a significant impact on the metabolic level and intestinal microbiota of the body [35]. This study comprehensively evaluated the impacts of SYD, *B. subtilis*, and SYD fermentation broth on rats’ intestinal microbiota. The results showed that SYD fermentation broth promoted the growth of intestinal probiotics and symbiotic bacteria to a certain extent compared with other groups and inhibited the propagation of pathogenic bacteria. These findings helped us to understand how changes in homeostasis caused by changes in the diversity of intestinal microbiota had a significant impact.

*Lactobacillus*, an important probiotic in the animal husbandry industry, has been widely recognized by people in the industry for its beneficial effects on animal organisms [36]. Lactobacillus can be used to treat intestinal infections caused by bacteria, stimulate the body’s humoral and cellular immunity, reduce inflammation, promote gastrointestinal motility, prevent depression, and relieve lactose intolerance [37–39]. The results of our research showed that Lactobacillus is the most important genus among all intestinal microbiota. Similar to Lactobacillus, Bifidobacterium is an important physiological bacterium found in human and animal intestine. It plays an important role in a series of physiological processes, such as immunity [40], nutrition [41], digestion [42], and protection [43]. The importance of *Bifidobacterium* to human health can be recognized from its early colonization in the neonatal gut, where *Bifidobacterium longum* is the most abundant species. The beneficial properties of *B*. *longum* have been revealed through a series of mechanisms, including the production of bioactive molecules, such as short-chain fatty acids, polysaccharides, and serine protease inhibitors [44].

Studies have shown that TCM can promote the growth and reproduction of probiotics and symbiotic bacteria and reduce the increase in the number of related pathogens, thereby playing a positive regulatory role in the intestinal microbiota of animals [45]. Moreover, TCM or functional foods fermented with probiotics have significantly improved their functional effects and have played a supplementary role in animal health [46]. The number of probiotics was significantly higher in the fermented group than in the unfermented and control groups in our study.

Some pathogens, including *Escherichia coli* and others, were observed in the samples of this study. These microorganisms act as opportunistic pathogens in the intestine, but, like *E. coli*, they require a series of unbalanced conditions or certain serotypes to cause symptoms harmful to animal health such as diarrhea. It has an important influence on the growth, development, and production performance of animals [47]. Although the numbers of these microbiota in the four experimental groups were small and the differences were not significant, they deserve close attention. Therefore, in future work, we believe that it is necessary to strengthen the observation and analysis of these microbiota.

For the first time in this study, the intestinal microbiota of rats fed SYD fermentation broth were analyzed using high-throughput sequencing technology. However, some inevitable errors have occurred in sample sequencing, bioinformatics analysis, and library preparation. Improvement of experimental methods can minimize the occurrence of experimental errors [48, 49]. In addition, the lack of 16S rRNA gene sequences in existing databases hindered the development of sequencing technology [50]. Although some common microbiota in rat fecal samples were analyzed in this study, many strains still require further classification and functional identification.

In conclusion, 16S rRNA-based microbiological analysis of the intestinal microbiota of rats fed different diets was performed using high-throughput Illumina sequencing technology. Intestinal microbiota structure of rats changed considerably after different treatments. Although the bacterial community is affected by many factors and conditions, the structural characteristics of the fecal bacterial communities in each treatment group were as detailed as possible. SYD fermentation broth and *B. subtilis* significantly improved the balance of microbiota, increased the number of probiotics, and inhibited the growth of pathogens. However, the mechanism by which SYD affects the internal balance of intestinal microbiota remains unclear. Therefore, further transcriptome and metabolomics studies and related molecular biological validation are required to fully understand the potential regulatory mechanisms of specific functional intestinal microbiota associated with animals.

## Conclusion

After fermentation, the effect of SYD was significantly better than that of SYD or *B. subtilis*. SYD significantly promoted the growth of intestinal probiotics, inhibited the growth of pathogenic bacteria, and maintained the balance of intestinal microbiota in SD rats. This study provides new insights into the development and use of SYD.

## Authors’ Contributions

ZY, KC, YL, XW, and BH: Collected the samples and designed and supervised the study. KC and BH: Data collection and analysis. KC, SW, and BH: Conceived the idea of the study and drafted and edited the manuscript. All authors have read, reviewed, and approved the final manuscript.
